# Differences in Volatile Organic Compounds in *Rhizoma gastrodiae* (Tian Ma) of Different Origins Determined by HS-GC-IMS

**DOI:** 10.3390/molecules28134883

**Published:** 2023-06-21

**Authors:** Hao Duan, Yaxi Zhou, Diandian Wang, Wenjie Yan

**Affiliations:** 1 College of Biochemical Engineering, Beijing Union University, Beijing 100023, China; dhuanao@163.com (H.D.);; 2 Beijing Key Laboratory of Bioactive Substances and Functional Food, Beijing Union University, Beijing 100023, China

**Keywords:** *Rhizoma gastrodiae*, HS-GC-IMS, identification, different origins, cluster analysis

## Abstract

Headspace gas chromatography–ion mobility spectrometry (HS-GC-IMS) and principal component analysis (PCA) were used to compare the differences in volatile organic compounds (VOCs) of *Rhizoma gastrodiae* (Tian Ma) from six different origins in Yunnan, Sichuan, Shaanxi, Anhui, Hubei, and Guizhou. A total of 161 signal peaks were identified, and 84 compounds were characterized, including 23 aldehydes, 19 alcohols, 12 ketones, 8 heterocyclic compounds, 7 esters, 4 phenols, 4 acids, 4 ethers, 2 amines, and 1 alkane. The results of cluster analysis and fingerprint similarity analysis based on principal component analysis and Euclidean distance indicated that there were significant differences between the volatile components of *Rhizoma gastrodiae* from different origins. This study demonstrated that HS-GC-IMS is simple, rapid, accurate, and has a small sample size and can achieve rapid analysis of the differences in volatile compounds between samples of different origins of *Rhizoma gastrodiae*.

## 1. Introduction

*Rhizoma gastrodiae* (also known as Tian ma) is mainly produced in Asian countries such as China, Korea, Japan, and India [1]. In areas such as Guizhou, Yunnan, Hunan, Sichuan, and Shannxi in China, *Rhizoma gastrodiae* is used as food in stews, hot pots, and stir-fries by the inhabitants of these regions [2]. Studies have shown that there are significant differences in chemical composition and pharmacological activity between different origins of *Rhizoma gastrodiae* [3]. In Asia, *Rhizoma gastrodiae* is used both as a food and medicinal plant. In China, Gastrodia elata is recommended for treating headaches, dizziness, tremors, and epilepsy, and to help improve central nervous system disorders. Modern research has also confirmed that *Rhizoma gastrodiae* has shown good application in central nervous system disorders such as Alzheimer’s disease, epilepsy, Parkinson’s disease, cerebral ischemia/reperfusion, and cognitive dysfunction [4,5,6]. The main active ingredients of *Rhizoma gastrodiae* to improve central nervous system (CNS) disorders are its rich phenolic compounds, such as gastrodin, p-hydroxybenzyl alcohol, and parishin [7]. In addition, *Rhizoma gastrodiae* is also rich in polysaccharides, sterols, organic acids, and other components, which constitute the special smell of *Rhizoma gastrodiae* [7], and it is rich in volatile components [8]. In recent years, *Rhizoma gastrodiae* has gained much attention and popularity due to its better edible and medicinal value. It is being processed into relevant functional foods and occupys a certain proportion of consumption in the market, and volatile flavor compounds play an important role in the acceptability of *Rhizoma gastrodiae* products, which has undoubtedly aroused great interest in the study of *Rhizoma gastrodiae* flavor.

At present, there are two parts to the analysis of volatile substances in food: sensory analysis, which is a kind of sensory perception and evaluates the results with a certain subjectivity, and instrumental analysis, which is an objective analysis at the molecular level, and the combination of these two forms in order to evaluate the flavors of food more scientifically [9]. As a result, there has been an increasing amount of research in recent years on the use of instrumental analysis techniques to detect volatile flavor components in food products. The commonly used instrumental analytical techniques for volatile compounds in food are gas chromatography–mass spectrometry (GC-MS), gas chromatography–ion mobility spectrometry (GC-IMS), gas chromatography olfaction–mass spectrometry (GC-O-MS), and electronic nose (E-nose). Although E-nose has the advantage of being fast and having sensitive detection, the reproducibility of the results is somewhat lower and, therefore, electronic noses are limited in practical application [10,11]. GC-IMS has been shown to be fast, sensitive, and easy to operate [9], and compared to GC-MS, the former has a higher separation efficiency and can be used to obtain analytical results in a shorter period of time. In recent years, GC-IMS and GC-MS have been widely used for the study of volatile compounds in traditional Chinese medicine and foodstuffs [12], such as Cordyceps sinensis, [13] green tea [14], and thuja [15]. Zhang et al. [16] established a rapid and accurate method for the determination of volatile components using gas chromatography–ion mobility spectrometry (GC-IMS) and gas chromatography–mass spectrometry (GC-MS). Guo et al. [17] used GC-MS and GC-IMS to analyze the aroma characteristics of oolong tea made from three tea varieties. However, few contemporary studies using HS-GC-IMS have been conducted on *Rhizoma gastrodiae*, and comprehensive studies on multiple origins of dried *Rhizoma gastrodiae* have not been reported.

Research has shown that flavors determine the organoleptic value of foods and also play an important role in identifying the nutritional value of foods. The richest source of *Rhizoma gastrodiae* is mainly in China, but the existence of differences in volatile flavors substances between multiple origins of dried *Rhizoma gastrodiae* has not been explored in current studies. Therefore, in the current study, for comparison, we compared the differences in volatile organic matter of *Rhizoma gastrodiae* from six origins: Yunnan, Sichuan, Shaanxi, Anhui, Hubei, and Guizhou. The volatile flavor substances identified in this study can provide some reference value for the processing and nutritional value of *Rhizoma gastrodiae* functional foods.

## 2. Results

### 2.1. Spectral Analysis of Rhizoma gastrodiae Samples from Different Origins

**Figure 1:**
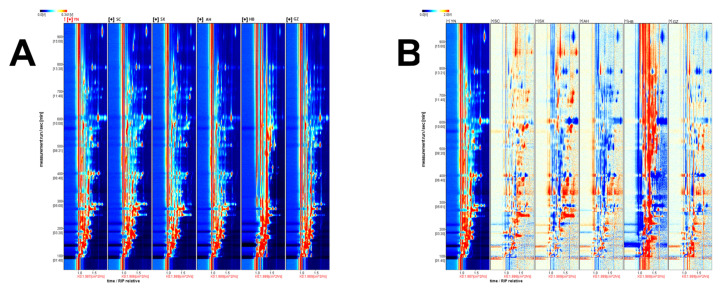
Topographical map of the different origins of *Rhizoma gastrodiae* (**A**) and comparative map of sample differences (**B**).

HS-GC-IMS was used to analyze the VOCs in six *Rhizoma gastrodiae* samples. A two-dimensional top view of the VOCs in the six *Rhizoma gastrodiae* samples was plotted using the Reporter plug-in (Figure 1), enabling a detailed comparison of the differences in VOCs between the different *Rhizoma gastrodiae* samples. The vertical coordinates indicate the retention time of the GC, and the horizontal coordinates indicate the drift time and the reactive ion peak (RIP). The different colored dots to the right of the RIP represent the different VOCs detected. The difference in color reflects the signal intensity of the different volatiles detected in each *Rhizoma gastrodiae* sample, with the red signal indicating a higher concentration of the detected volatiles and, the darker the color, the greater the intensity, indicating a greater concentration of that volatile substance and vice versa. As can be seen in Figure 1A, most of the signal occurs at retention times of 80–970 s and drift times of 1.0–1.9 ms.

A differential contrast model was used to compare the differences between samples, using the YN sample as the reference contrast and the remaining sample minus the reference. If the two VOCs agree, the background after subtraction is white; if it is red, it means that the substance concentration is higher than the reference, and blue means that it is lower than the reference. In the differential contrast model plot (Figure 1B), the concentrations of the volatile substances can be clearly seen. Comparing samples YN, SC, and SX, it can be seen that in the range of retention times of 190–270 s and drift times of 1.4–1.8 ms, the concentrations of petanal, hexanal, pentan-1-ol, methyl isobutyl ketone, 2-hexanone, and butanoic acid in SC and SX methyl esters and other VOC concentrations were significantly higher than YN. Comparing samples YN, SX, and AH, it can be found that the VOC concentrations of heptanal, 2-heptanone, n-hexanol, styrene, 1,2-dimethylbenzene, and pentanoic acid in SX and AH were significantly higher and were in the retention time range of 350–400 s and 1.0–2.0 ms. Comparing samples YN and HB, it can be found that in the drift time range of 1.0–1.7 ms, the concentrations of 5-methylfurfural, butanoic acid methyl eater, 3-hydroxybutan-2-one, alpha-pinene, 1-heptanol, and ethyl hexanoate in HB were significantly higher than YN. Heptanol, ethyl hexanoate, 3-octanol, 2-methylbutan-1-ol, N, N-diethylethanamine, isopropyl acetate, octan-1-ol, diethylene glycol dimethyl ether, alpha-phellandrene, 2,6-dimethylphenol, 2-penthlfuran, and other VOCs were significantly higher than in YN. However, comparing samples YN and GZ, it can be seen that the concentration of volatile components in the GZ sample is significantly lower than YN. These differences in data indicate that the differences in climate, soil, environment, and seed quality among different regions have led to differences in volatile components in *Rhizoma gastrodiae* from each production area. This also indirectly indicates that under the current experimental conditions, compared with other regions, the *Rhizoma gastrodiae* from Hubei contains more flavorful substances, which explains why it has a more obvious aroma in all the samples.

### 2.2. Identification of Volatile Components in Different Rhizoma gastrodiae Samples

Lu et al. [18] analyzed and identified the volatile flavor components in *Rhizoma gastrodiae* and mainly included alkanes (16.63%), alkenes (10.14%), aldehydes (57.06%), alcohols (3.62%), esters (6.38%), ketones (1.04%), heterocyclic compounds (1.07%), and ethers (0.32%). Sun et al., used GC-IMS to analyze the differences in volatile compounds in fresh *Rhizoma gastrodiae* from different origins, and a total of 75 volatile compounds were detected. Of these, 45 substances could be identified, including 16 aldehydes, 9 esters, 6 alcohols, 5 ketones, and 3 acids [19].

**Figure 2:**
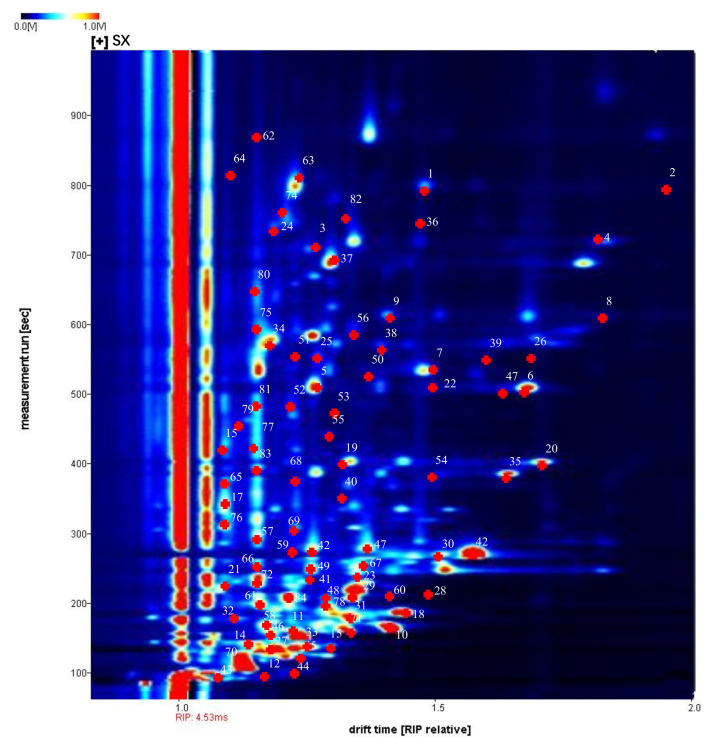
HS-GC-IMS profiles of *Rhizoma gastrodiae* samples from different origins. The numbers in the graph indicate the volatile components identified.

In this study, the differences in volatile organic compounds in *Rhizoma gastrodiae* from 6 origins in Yunnan, Sichuan, Shaanxi, Anhui, Hubei, and Guizhou were analyzed using HS-GC-IMS, and a total of 161 signal peaks were identified. These compounds were characterized by comparing their IMS drift times and retention indices with authentic reference compounds. A total of 84 typical target compounds were identified from the topography by the GC × IMS library. As shown in Figure 2, the horizontal coordinates indicate the drift time, the vertical coordinates indicate the retention time, and the white numbers correspond to the compounds in Table 1. A total of 84 compounds were characterized, including 23 aldehydes, 19 alcohols, 12 ketones, 8 heterocyclic compounds, 7 esters, 4 phenols, 4 acids, 4 ethers, 2 amines, and 1 alkane compound. Of these, there were nonanal, (*E*)-hept-2-enal, octanal, 3-methylbutanal, hexanal, butanal, heptanal, (*E*)-2-pentenal, 1-octen-3-one, 3-octanol, isopropyl alcohol, alpha-pinene, and ortho-guaiacol. These 13 compounds were present as monomers and dimers. The method appears to be able to detect a wider range of organic compounds with richer results than the conventional GC-IMS analysis technique.

Previously, Sun et al., analyzed the differences in volatile compounds in fresh *Rhizoma gastrodiae* of three varieties using GC-IMS, and a total of 75 volatile compounds were detected, including 45 identified substances such as aldehydes, esters, alcohols, ketones, and acids [19]. Our study also found that aldehydes were the highest substance in the volatile components of *Rhizoma gastrodiae*, and our method using HS-GC-IMS could detect more abundant volatile components. We speculated that this may be due to the more abundant volatile components contributed by Hubei *Rhizoma gastrodiae*.

### 2.3. Topographic Results and Analysis of Rhizoma gastrodiae Samples from Different Origins

**Figure 3:**
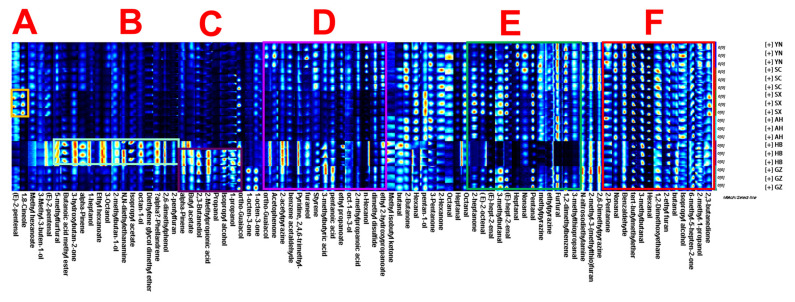
Fingerprints of samples of *Rhizoma gastrodiae* from different origins.

In order to better compare the differences in specific substances in each group of samples, the peaks of all *Rhizoma gastrodiae* samples were selected for comparison in gallery plot fingerprinting (Figure 3). Each row in Figure 3 represents the substance detected, and each column represents the signal intensity of the same volatile substance in the different *Rhizoma gastrodiae* samples. Each dot represents a volatile substance, and the shade of color reflects the level of volatility in that sample; the brighter the color, the higher the level. The opposite is also true. Of these, 2-pentanone, nonana, benzaldehyde, tert-butylmethylether, 3-methylbutanal, hexanal, 1,2-dimethoxyethane, 2-ethyl furan, butanal isopropyl alcohol, 6-methyl-5-hepten-2-one, 2-methyl-1-propanol, and 2,3-butanedione showed essentially no differences between all samples of *Rhizoma gastrodiae* (Box F).

Characteristic substances of 1,8-cineole and (*E*)-2-pentenal Shaanxi *Rhizoma gastrodiae* (Box A).

5-methylfurfural, butanoic acid methyl eater, 3-hydroxybutan-2-one, alpha-pinene, 1-heptanol, ethyl hexanoate, 3-octanol, 2-methylbutan-1-ol, N,N-diethylethanamine, isopropyl acetate, octan-1-ol, diethylene glycol dimethyl ether, alpha-phellandrene, 2,6-dimethylphenol, and 2-penthlfuran are characteristic substances of Hubei *Rhizoma gastrodiae* (Box B).

Alpha-pinene, butyl acetate, 2,3-butanediol, 2-methylpropionic acid, propanal, isopropyl alcohol, 1-propanol, and ortho-guaiacol are characteristic substances of *Rhizoma gastrodiae* from Hubei and Guizhou (Box C).

Ortho-guaiacol, acetophenone, 2-acetylpyrazine, benzene acetaldehyde, pyridine,2,4,6-trimethyl, furaneol, styrene, 3-methylbutyric acid pentanoic acid, ethyl propanoate, oct-1-en-3-ol, 2-methylpropanoic acid, n-hexanol, dimethyl disulfide, and ethyl 2-hydroxypropanoate were the characteristic substances of the samples, except for those from Shaanxi and Anhui, which were characteristic substances of the samples (Box D).

2-heptanone, (*E*)-2-octenal, v-hept-2-enal, 3-methylbutanal, heptanal, nonanal, pentanal, methylpyrazine, ethylpyrazine, furfural, 1,2-dimethylbenzene, and 3-methylthiopropanal are characteristic of origins other than Hubei (Box E).

The above results indicate that the volatile components contained in *Rhizoma gastrodiae* from different origins vary more significantly and can be distinguished from those of different origins by HS-GC-IMS.

### 2.4. Cluster Analysis of Volatile Components in Samples of Rhizoma gastrodiae from Different Origins

#### 2.4.1. Dynamic PCA of Samples

**Figure 4:**
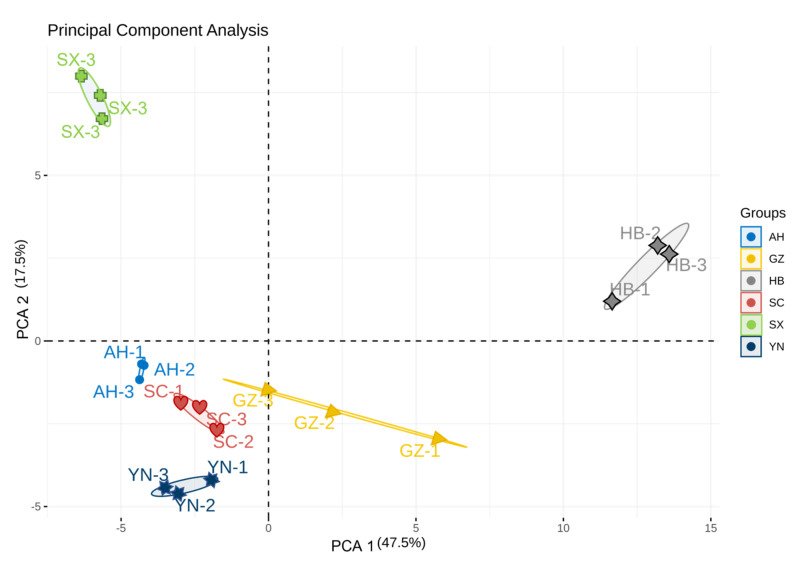
Results of the PCA analysis of *Rhizoma gastrodiae* samples from different origins.

Principal component analysis (PCA) is a multivariate statistical method used to examine correlations among multiple variables [20]. PCA serves as a powerful visualization tool that provides researchers with a way to reduce the dimensionality of data, thereby eliminating non-essential information [21,22]. In this study, a PCA analysis was performed on *Rhizoma gastrodiae* samples, and the results are shown in Figure 4, where different colors represent different samples of *Rhizoma gastrodiae*, the distance between individual points represents the level of similarity, and the dispersion of the same points represents the homogeneity of the same sample. The PCA (Figure 4) illustrates the differences in the contribution of different volatile flavor substances to different samples. When samples are closely located, it can be understood that the differences in flavor compounds between samples are relatively small. Conversely, it indicates a significant difference in volatile components between the two. According to Figure 4, Dim1 accounts for 47.5% and Dim2 accounts for 17.5%, with a total cumulative contribution of 65% by the two principal components. Figure 4 also illustrates significant differences in aroma components of *Rhizoma gastrodiae* from different production areas. *Rhizoma gastrodiae* from Anhui, Sichuan, and Yunnan are grouped together, while Shaanxi and Hubei are significantly different than other production areas, and there are also significant differences between *Rhizoma gastrodiae* samples from the three production areas. This indicates that HS-GC-IMS data contains effective information that can distinguish *Rhizoma gastrodiae* samples from different production origins.

#### 2.4.2. Fingerprint Similarity Analysis Based on Euclidean Distance

**Figure 5:**
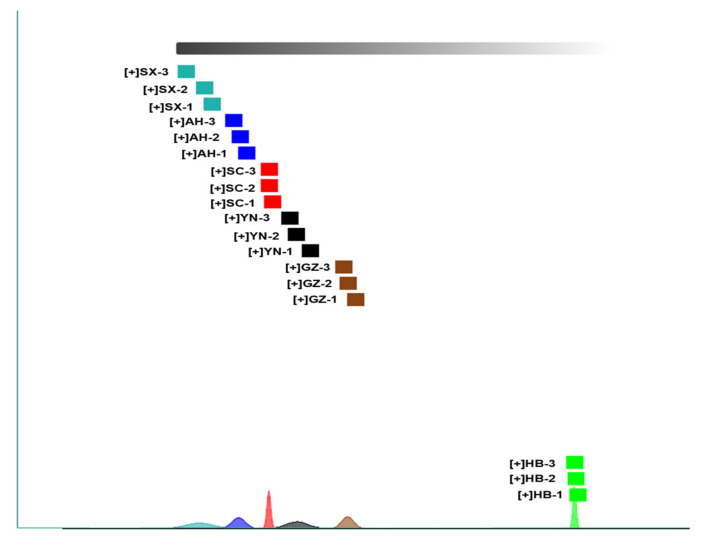
Fingerprint similarity based on Euclidean distance of different samples.

Euclidean distance, similar to PCA analysis, is a method of cluster analysis in which similarity is determined by a distance coefficient; if the coefficient is large, the difference between the two is also large and shows a positive correlation. Conversely, the smaller the coefficient, the smaller and more similar the difference between the two [23]. By applying the Euclidean distance similarity algorithm, it is possible to evaluate the quality of two samples, and it has been found that the algorithm can accurately and reliably evaluate the quality of samples. [23]. Figure 5 shows the fingerprint similarity based on Euclidean distance and Table S1 represents the Euclidean distance values between the samples of different origins of *Rhizoma gastrodiae*. The results of the Euclidean distance analysis show that the distances between the *Rhizoma gastrodiae* samples of different origins can be clearly distinguished. Among them, the average Euclidean distance between SX and AH was 6,257,676, the average Euclidean distance between AH and SC was 5,688,077, the average Euclidean distance between SC and YN was 6,284,151.444, the average Euclidean distance between YN and GZ was 8,169,782, the average Euclidean distance between GZ and HB was 23,577,777.778, and the average Euclidean distance between HB and SX was 34,733,333.333. So, HB and SX are the furthest apart, and the difference between them is considered to be the most significant.

#### 2.4.3. Hierarchical Cluster Analysis Heatmap

**Figure 6:**
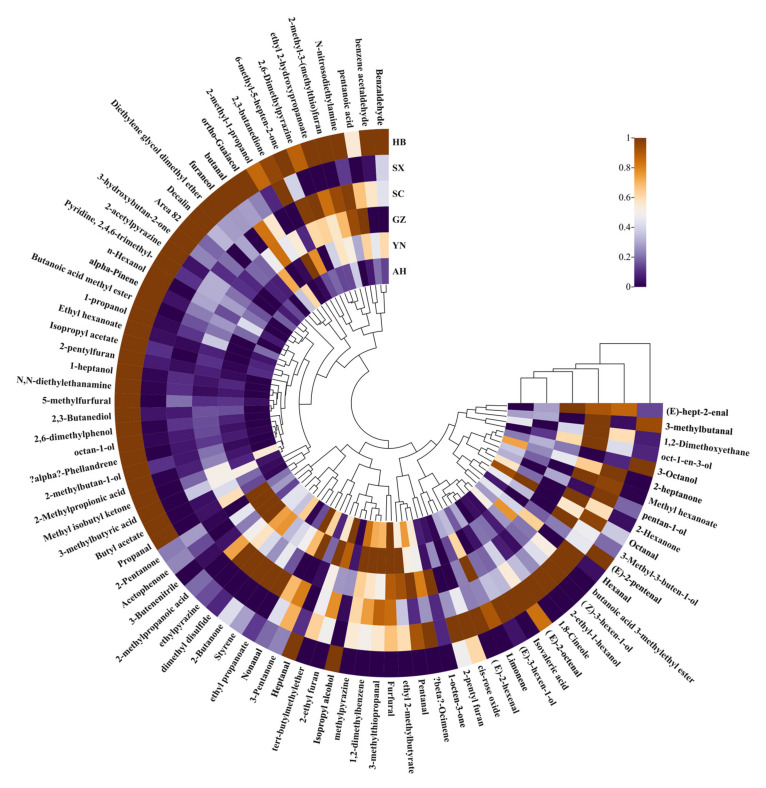
Hierarchical clustering heat map of volatile fractions identified in different *Rhizoma gastrodiae* samples.

To further analyze the differences in VOCs between different *Rhizoma gastrodiae* samples, a hierarchical cluster analysis (HCA) heatmap was generated, which can be used to distinguish between two main clusters [24] and is an important method of cluster analysis [25]. It has been widely used to analyze the degree of variation in food composition [26,27]. Figure 6 shows the HCA of volatiles in different *Rhizoma gastrodiae* samples. The outer circle represents the volatiles detected, and the contents of the column at the opening of the circle indicate the name of each *Rhizoma gastrodiae* sample. Purple indicates low relative intensity, while brown indicates high relative intensity, and the darker the color, the greater the intensity, and vice versa. It is evident in Figure 6 that the relative content of volatile substances varied between the samples, with the HB (Hubei *Rhizoma gastrodiae*) sample containing a higher and more diverse range of volatile substances, which was significantly different from the other Tianma samples. The other samples also showed some differences in the volatile substances of *Rhizoma gastrodiae*. Previous studies have shown that the conventional GC-MS technique is usually used to analyze VOCs and thus distinguish fresh *Rhizoma gastrodiae* from different origins and varieties [19]. In contrast, this study used HS-GC-IMS to analyze VOCs in *Rhizoma gastrodiae* samples from different origins, which had many advantages over the traditional GC-MS method such as more convenient operation, faster response, higher sensitivity, and lower cost.

## 3. Materials and Methods

### 3.1. Sample Preparation

**Figure 7:**
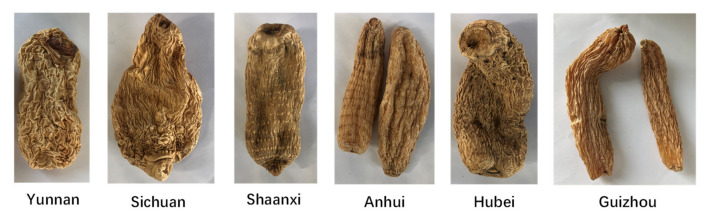
Samples of six species of *Rhizoma gastrodiae*.

The samples of the six species are detailed in Figure 7; each sample was crushed using a pulverizer (a high-speed crusher (model: FW80, produced by Tianjin, Tianjin, China, Tianjin Tasty Instruments Co., Ltd.)) and was further passed through a 24-mesh sieve for HS-GC-IMS analysis, and the processing information for each sample is given in Table 2.

### 3.2. HS-GC-IMS System

We used the Gas-phase Ion Mobility Spectrum Flavour Spec^®^ (G.A.S. department of Shandong Province, Qingdao, China, Shandong Hai Neng Science Instrument Co., Ltd.) to analyze 6 different regions of *Rhizoma gastrodiae* powder prepared previously. We placed 2 g of *Rhizoma gastrodiae* powder in a 20 mL top-empty bottle and incubator for 20 min at 70 °C and 500 rpm under gas phase temperature. Next, set the temperature of the injection needle to 85 °C and inject 300 microliters of the sample.

Then, perform gas chromatography separation using an MXT-5 chromatography column (15 m × 0.53 mm × 1 μm) at a column temperature of 60 °C. Set the IMS temperature to 45 °C and use N_2_ (purity ≥99.999 %) as the carrier/drift gas with a flow rate of 2 mL/min (0–2 min), 10 mL/min (2–10 min), 100 mL/min (10–20 min), 150 mL/min (20–30 min), and stop the analysis after 30 min. The drift tube is maintained at 45 °C under the N_2_ drift gas with a flow rate of 150 mL/min. Three samples are measured for each sample.

### 3.3. Data Analysis

GC-IMS library Search software (Version number: 1.0.3) and the Laboratory Analytical Viewer (LAV) are data analysis software (Version number: 2.2.1) that allow different perspectives to be examined. The LAV includes VOCal and three plug-ins, and VOCal is used to view analytical spectra and qualitative and quantitative analysis data. Volatile organic compounds are represented by each point on the graph. With the software’s built-in database, qualitative analyses of substances can be performed. A Reporter plug-in can be used to compare the spectral differences between different products, such as 2D top views and sample difference spectra. Using the library plot plug-in, differences in VOCs between the samples were visually compared using inter-sample fingerprinting; in order to facilitate rapid identification of unknown sample types, the dynamic PCA plug-in was used for cluster analysis of the samples. A principal component analysis was used to investigate the relationship between different samples and VOCs; using the clustering heat map tool, heat maps were created.

## 4. Conclusions

In this study, headspace gas chromatography–ion mobility spectrometry (GC-IMS) and principal component analysis (PCA) were used to compare the differences in volatile organic compounds in *Rhizoma gastrodiae* from six origins in Yunnan, Sichuan, Shaanxi, Anhui, Hubei, and Guizhou. A total of 161 signal peaks were identified, and 84 compounds were characterized, including 23 aldehydes, 19 alcohols, 12 ketones, 8 heterocyclic compounds, 7 esters, 4 phenols, 4 acids, 4 ethers, 2 amines, and 1 alkane compound. Due to the limitations of the HS-GS-IMS assay and the fact that 77 signal peaks have not yet been identified, further qualitative analysis can be carried out at a later stage by other analytical techniques, such as HPLSMS (High-Performance Liquid Chromatography–Mass Spectrometry).

The results of the cluster analysis and fingerprint similarity analysis based on the principal component analysis, as well as Euclidean distance, showed that *Rhizoma gastrodiae* mainly contained 2-pentanone, nonana, benzaldehyde, tert-butylmethylether, 3-methylbutanal, hexanal, 1,2- dimethoxyethane, 2-ethyl furan, butanal, isopropyl alcohol, 6-methyl-5-hepten-2-one, 2-methyl-1-propanol, and 2,3-butanedione, with some variation between different origins of *Rhizoma gastrodiae*. The higher content and variety of volatile substances were contained in the HB (Hubei *Rhizoma gastrodiae*) samples. This indicates that the differences between different origins influenced the results of the detection of volatile substances in *Rhizoma gastrodiae*. It also shows that under the present experimental conditions, the quality of Hubei *Rhizoma gastrodiae* is better compared to other origins and varieties of *Rhizoma gastrodiae*.

In summary, we used HS-GC-IMS to perform three parallel tests on *Rhizoma gastrodiae* samples from each region. The operation is simple, fast, accurate, and requires a small sample size. Our study demonstrates that through the multivariate data analysis method of HS-GC-IMS, it is possible to analyze and distinguish *Rhizoma gastrodiae* from different geographic sources.

## Figures and Tables

**Table 1:** Results of the qualitative analysis of *Rhizoma gastrodiae* samples from different origins.

No.	Category	Compound	CAS#	Formula	MW	RI	Rt [sec]	Dt [a.u.]	Odors	Identification
1	Aldehydes	Nonanal	124-19-6	C9H18O	142.2	1085.1	789.724	1.47237	Fat, Floral, Green, Lemon	RI, Dt
2		Nonanal	124-19-6	C9H18O	142.2	1084.7	788.781	1.94538		RI, Dt
3		Benzene acetaldehyde	122-78-1	C8H8O	120.2	1051	710.027	1.26844	Berry, Geranium, Honey, Nut, Pungent	RI, Dt
4		(*E*)-2-octenal	2548-87-0	C8H14O	126.2	1052.3	712.857	1.81651	Dandelion, Fat, Fruit, Grass, Green, Spice	RI, Dt
5		(*E*)-hept-2-enal	18829-55-5	C7H12O	112.2	958.5	510.569	1.26618	Almond, Fat, Fruit	RI, Dt
6		(*E*)-hept-2-enal	18829-55-5	C7H12O	112.2	957.6	508.71	1.67657		RI, Dt
7		Benzaldehyde	100-52-7	C7H6O	106.1	970.9	533.909	1.49172	Bitter Almond, Burnt Sugar, Cherry, Malt, Roasted Pepper	RI, Dt
8		Octanal	124-13-0	C8H16O	128.2	1008.5	610.474	1.81899	Citrus, Fat, Green, Oil, Pungent	RI, Dt
9		Octanal	124-13-0	C8H16O	128.2	1007.9	608.849	1.40513		RI, Dt
10		3-methylbutanal	590-86-3	C5H10O	86.1	657.3	171.351	1.40132	/	RI, Dt
11		3-methylbutanal	590-86-3	C5H10O	86.1	648.2	167.358	1.20211	/	RI, Dt
12		Propanal	123-38-6	C3H6O	58.1	500.8	102.5	1.16456	Solvent, Pungent	RI, Dt
13		Hexanal	66-25-1	C6H12O	100.2	792.9	272.963	1.57113	Grass, Tallow, Fat	RI, Dt
14		butanal	123-72-8	C4H8O	72.1	607.8	149.594	1.13744	Pungent, Green	RI, Dt
15		3-methylthiopropanal	3268-49-3	C4H8OS	104.2	914.1	426.711	1.08638	Cooked potato, Soy	RI, Dt
16		Butanal	123-72-8	C4H8O	72.1	598.1	145.325	1.29128	Pungent, Green	RI, Dt
17		Furfural	98-01-1	C5H4O2	96.1	847.3	334.536	1.08819	Bread, Almond, Sweet	RI, Dt
18		Pentanal	110-62-3	C5H10O	86.1	695.9	191.106	1.43012	Almond, Malt, Pungent	RI, Dt
19		Heptanal	111-71-7	C7H14O	114.2	902.2	404.278	1.68518	Fat, Citrus, Rancid	RI, Dt
20		Heptanal	111-71-7	C7H14O	114.2	901.8	403.389	1.31849		RI, Dt
21		(*E*)-2-pentenal	1576-87-0	C5H8O	84.1	748.2	233.774	1.36115	/	RI, Dt
22		5-methylfurfural	620-02-0	C6H6O2	110.1	960.9	515.113	1.49172	Almond, Caramel, Burnt Sugar	RI, Dt
23		(*E*)-2-pentenal	1576-87-0	C5H8O	84.1	750.3	235.422	1.0914	/	RI, Dt
24	Ketones	Acetophenone	98-86-2	C8H8O	120.2	1060.7	732.663	1.18771	Must, Flower, Almond	RI, Dt
25		1-octen-3-one	4312-99-6	C8H14O	126.2	981.7	554.357	1.26719	Earth, Mushroom	RI, Dt
26		1-octen-3-one	4312-99-6	C8H14O	126.2	980.6	552.292	1.68896		RI, Dt
27		2,3-butanedione	431-03-8	C4H6O2	86.1	590.3	141.883	1.1849	Butter, Pastry, Yeast	RI, Dt
28		Methyl isobutyl ketone	108-10-1	C6H12O	100.2	731.4	220.04	1.48223	/	RI, Dt
29		3-hydroxybutan-2-one	513-86-0	C4H8O2	88.1	719.5	210.334	1.3328	Butter, Creamy, Green Pepper	RI, Dt
30		2-hexanone	591-78-6	C6H12O	100.2	792.8	272.779	1.50369	/	RI, Dt
31		3-pentanone	96-22-0	C5H10O	86.1	691.6	187.621	1.33136	/	RI, Dt
32		2-pentanone	107-87-9	C5H10O	86.1	684.2	183.193	1.10824	/	RI, Dt
33		2-butanone	78-93-3	C4H8O	72.1	593.1	143.122	1.25217	Fragrant, Fruit, Pleasant	RI, Dt
34		6-methyl-5-hepten-2-one	110-93-0	C8H14O	126.2	991.7	573.154	1.17337	Citrus, Mushroom, Pepper, Rubber, Strawberry	RI, Dt
35		2-heptanone	110-43-0	C7H14O	114.2	892.7	386.287	1.63641	Blue Cheese, Fruit, Green, Nut, Spice	RI, Dt
36	Alcohols	Octan-1-ol	111-87-5	C8H18O	130.2	1065.1	743.038	1.46954	Bitter Almond, Burnt Matches, Fat, Floral	RI, Dt
37		1.8-cineole	470-82-6	C10H18O	154.3	1039.2	682.204	1.2911	Camphor, Cool, Eucalyptol, Mint	RI, Dt
38		3-octanol	589-98-0	C8H18O	130.2	998.8	587.535	1.77055	Citrus, Moss, Mushroom, Nut, Oil	RI, Dt
39		Oct-1-en-3-ol	3391-86-4	C8H16O	128.2	981	552.912	1.60058	/	RI, Dt
40		N-hexanol	111-27-3	C6H14O	102.2	867.6	357.413	1.31308	Banana, Flower, Grass, Herb	RI, Dt
41		2-methylbutan-1-ol	137-32-6	C5H12O	88.1	749.6	234.873	1.25846	Fish Oil, Green, Malt, Onion, Wine	RI, Dt
42		Hexanal	66-25-1	C6H12O	100.2	798	278.639	1.25846	Grass, Tallow, Fat	RI, Dt
43		Isopropyl alcohol	67-63-0	C3H8O	60.1	510.2	106.632	1.07642	Floral	RI, Dt
44		Isopropyl alcohol	67-63-0	C3H8O	60.1	510.2	106.632	1.22088		RI, Dt
45		1-propanol	71-23-8	C3H8O	60.1	551.5	124.808	1.25217	Alcohol, Candy, Pungent	RI, Dt
46		2-methyl-1-propanol	78-83-1	C4H10O	74.1	631.3	159.922	1.17551	Apple, Bitter, Cocoa, Wine	RI, Dt
47		2,3-butanediol	513-85-9	C4H10O2	90.1	798.1	278.823	1.36805	/	RI, Dt
48		3-methyl-3-buten-1-ol	763-32-6	C5H10O	86.1	721.5	211.982	1.28375	/	RI, Dt
49		Pentan-1-ol	71-41-0	C5H12O	88.1	768.6	250.332	1.51699	Balsamic, Fruit, Green, Pungent, Yeast	RI, Dt
50		1-heptanol	111-70-6	C7H16O	116.2	966.2	525.027	1.36746	/	RI, Dt
51		?Alpha?-phellandrene	99-83-2	C10H16	136.2	981.1	553.118	1.2278	Citrus, Fresh, Mint, Pepper, Spice, Wood	RI, Dt
52		Alpha-pinene	80-56-8	C10H16	136.2	940.9	477.314	1.30277	Cedarwood, Pine, Sharp	RI, Dt
53		Alpha-pinene	80-56-8	C10H16	136.2	946.1	487.022	1.21548		RI, Dt
54		Styrene	100-42-5	C8H8	104.2	891.9	384.954	1.50274	Balsamic, Gasoline	RI, Dt
55	Esters	Methyl hexanoate	106-70-7	C7H14O2	130.2	922	441.592	1.28598	Fruit, Fresh, Sweet	RI, Dt
56		Ethyl hexanoate	123-66-0	C8H16O2	144.2	1000	590.425	1.33592	Apple Peel, Fruit	RI, Dt
57		Ethyl 2-hydroxypropanoate	97-64-3	C5H10O3	118.1	815.4	298.417	1.15117	Cheese, Floral, Fruit, Pungent, Rubber	RI, Dt
58		Isopropyl acetate	108-21-4	C5H10O2	102.1	667	175.62	1.16195	Banana	RI, Dt
59		Butyl acetate	123-86-4	C6H12O2	116.2	800.5	281.569	1.22474	Apple, Banana	RI, Dt
60		Butanoic acid methyl ester	623-42-7	C5H10O2	102.1	729.8	218.758	1.4102	Apple, Banana, Cheese, Ester, Floral	RI, Dt
61		Ethyl propanoate	105-37-3	C5H10O2	102.1	714.1	205.939	1.15347	Apple, Pineapple, Rum, Strawberry	RI, Dt
62	Phenols	2,6-dimethylphenol	576-26-1	C8H10O	122.2	1116.5	863.291	1.14948	Cresol, Phenol	RI, Dt
63		ortho-guaiacol	90-05-1	C7H8O2	124.1	1092.3	806.701	1.22878	Burnt, Phenol, Wood	RI, Dt
64		Ortho-guaiacol	90-05-1	C7H8O2	124.1	1093.7	810.002	1.10416		RI, Dt
65		1,2-dimethylbenzene	95-47-6	C8H10	106.2	870.1	360.3	1.08819	/	RI, Dt
66	Acids	2-methylpropanoic acid	79-31-2	C4H8O2	88.1	779.7	259.411	1.14811	Burnt, Butter, Cheese, Sweat	RI, Dt
67		2-methylpropionic acid	79-31-2	C4H8O2	88.1	786.9	266.187	1.36345		RI, Dt
68		Pentanoic acid	109-52-4	C5H10O2	102.1	888.4	380.956	1.22998	Sweat	RI, Dt
69		3-methylbutyric acid	503-74-2	C5H10O2	102.1	829.9	314.768	1.22547	Cheese, Pungent	RI, Dt
70	Ether	Tert-butylmethylether	1634-04-4	C5H12O	88.1	546.8	122.743	1.11658	/	RI, Dt
71		1,2-dimethoxyethane	110-71-4	C4H10O2	90.1	650.7	168.459	1.31631	/	RI, Dt
72		Dimethyl disulfide	624-92-0	C2H6S2	94.2	753	237.62	1.14964	Onion, Cabbage, Putrid	RI, Dt
73		Diethylene glycol dimethyl Ether	111-96-6	C6H14O3	134.2	956.8	507.264	1.62506	/	RI, Dt
74	Heterocyclic	Furaneol	3658-77-3	C6H8O3	128.1	1066.9	747.282	1.58425	Caramel	RI, Dt
75		Pyridine, 2,4,6-trimethyl-	108-75-8	C8H11N	121.2	1001.3	593.602	1.15262	/	RI, Dt
76		Methylpyrazine	109-08-0	C5H6N2	94.1	825.7	310.103	1.08729	Cocoa, Green, Hazelnut, Popcorn, Roasted	RI, Dt
77		2,6-dimethylpyrazine	108-50-9	C6H8N2	108.1	914	426.489	1.1478	Cocoa, Coffee, Green, Roast Beef, Roasted Nut	RI, Dt
78		2-ethyl furan	3208-16-0	C6H8O	96.1	700.2	194.585	1.28835	Butter, Caramel	RI, Dt
79		Ethylpyrazine	13925-00-3	C6H8N2	108.1	930.9	458.473	1.11528	Burnt, Green, Iron Scorch, Must, Peanut Butter, Roasted, Rum, Wood	RI, Dt
80		2-acetylpyrazine	22047-25-2	C6H6N2O	122.1	1024.7	648.224	1.14491	Roast, Roasted Corn, Toasted Cereal	RI, Dt
81		2-methyl-3-(methylthio)furan	63012-97-5	C6H8OS	128.2	942.1	479.586	1.14873	Savory	RI, Dt
82	Alkanes	Decalin	91-17-8	C10H18	138.3	1068.2	750.112	1.33075	/	RI, Dt
83	Amines	N-nitrosodiethylamine	55-18-5	C4H10N2O	102.1	898.6	397.392	1.15051	/	RI, Dt
84		N,N-diethylethanamine	121-44-8	C6H15N	101.2	707.6	200.629	1.20942	/	RI, Dt

Note: MW: molecular mass; RI: retention index; Rt: retention time; Dt: drift time.

**Table 2:** Information on the handling of *Rhizoma gastrodiae* samples.

No.	Sample Origin	Description	Test No.
1	Yunnan	Many folds, rough grain, short length	YN
2	Sichuan	Fewer folds, rougher grain, shorter length	SC
3	Shaanxi	Few folds, smoother and shorter in length	SX
4	Anhui	Few folds, relatively smooth, medium length	AH
5	Hubei	Many folds, rough grain, short length	HB
6	Guizhou	Fewer folds, rougher grain, longer length	GZ

## Data Availability

Data are contained within the article and Supplementary Materials.

## Supplementary Materials

The following supporting information can be downloaded at https://www.mdpi.com/article/10.3390/molecules28134883/s1, Table S1: Euclidean distances between different *Rhizoma gastrodiae* species.

## Sample Availability

Samples of the compounds are not available from the authors.
