# Supplementary material for: Differences in Volatile Organic Compounds in Rhizoma gastrodiae (Tian Ma) of Different Origins Determined by HS-GC-IMS
Source: Molecules. 2023 Jun 21;28(13):4883. doi: 10.3390/molecules28134883 (PMC10343826; doi:10.3390/molecules28134883)
Supplement: Supplementary file 1 [file molecules-28-04883-s001.zip › molecules-2403059-supplementary.pdf]

Table S1. Euclidean distances between different *Rhizoma Gastrodiae* species.

Full distance	[+] YN-1	[+] YN-2	[+] YN-3	[+] SC-1	[+] SC-2	[+] SC-3
[+] YN-1	0	1861900	1294329	6262191	6078085	6841483
[+] YN-2	1861900	0	1365138	5513500	6493920	5891848
[+] YN-3	1294329	1365138	0	6601739	6014513	6860084
[+] SC-1	6262191	5513500	6601739	0	1570583	1455731
[+] SC-2	6078085	6493920	6014513	1570583	0	904198.9
[+] SC-3	6841483	5891848	6860084	1455731	904198.9	0
[+] SX-1	6919033	7888214	7448222	6154776	5968429	6113464
[+] SX-2	7692156	8319867	8047045	7183915	6521240	6130134
[+] SX-3	7977968	8179582	8163960	6632007	7153707	6937454
[+] AH-1	5275692	6443629	5105756	7012095	5127716	6260915
[+] AH-2	5267957	5634279	5260557	6326016	4958909	5291033
[+] AH-3	4641406	4661386	4537810	5112211	5210326	5893472
[+] HB-1	2.80E+07	2.82E+07	2.93E+07	2.73E+07	2.56E+07	2.52E+07
[+] HB-2	3.02E+07	3.05E+07	3.17E+07	3.04E+07	2.93E+07	2.89E+07
[+] HB-3	3.15E+07	3.19E+07	3.29E+07	3.18E+07	3.02E+07	2.98E+07
[+] GZ-1	1.07E+07	1.21E+07	1.29E+07	1.21E+07	1.10E+07	1.13E+07
[+] GZ-2	6238753	7117121	8362659	7008866	6438451	6067624
[+] GZ-3	4353690	6001100	5754715	6084378	4955168	5329886

Cont.

Full distance	[+] SX-1	[+] SX-2	[+] SX-3	[+] AH-1	[+] AH-2	[+] AH-3
[+] YN-1	6919033	7692156	7977968	5275692	5267957	4641406
[+] YN-2	7888214	8319867	8179582	6443629	5634279	4661386
[+] YN-3	7448222	8047045	8163960	5105756	5260557	4537810

[+] SC-1	6154776	7183915	6632007	7012095	6326016	5112211
[+] SC-2	5968429	6521240	7153707	5127716	4958909	5210326
[+] SC-3	6113464	6130134	6937454	6260915	5291033	5893472
[+] SX-1	0	460129.2	546565.8	6105223	5897565	6090298
[+] SX-2	460129.2	0	553084.3	6141732	5965468	6599411
[+] SX-3	546565.8	553084.3	0	6673725	6560360	6285301
[+] AH-1	6105223	6141732	6673725	0	484778.7	1064562
[+] AH-2	5897565	5965468	6560360	484778.7	0	848686.1
[+] AH-3	6090298	6599411	6285301	1064562	848686.1	0
[+] HB-1	3.24E+07	3.22E+07	3.34E+07	3.10E+07	3.06E+07	3.16E+07
[+] HB-2	3.52E+07	3.50E+07	3.57E+07	3.49E+07	3.43E+07	3.47E+07
[+] HB-3	3.62E+07	3.57E+07	3.68E+07	3.59E+07	3.53E+07	3.61E+07
[+] GZ-1	1.60E+07	1.62E+07	1.69E+07	1.51E+07	1.47E+07	1.47E+07
[+] GZ-2	9133866	9499591	1.04E+07	8448925	7157028	7619833
[+] GZ-3	6103869	6415780	7412826	5004355	4523457	4898795

Cont.

Full distance	[+] HB-1	[+] HB-2	[+] HB-3	[+] GZ-1	[+] GZ-2	[+] GZ-3
[+] YN-1	2.80E+07	3.02E+07	3.15E+07	1.07E+07	6238753	4353690
[+] YN-2	2.82E+07	3.05E+07	3.19E+07	1.21E+07	7117121	6001100
[+] YN-3	2.93E+07	3.17E+07	3.29E+07	1.29E+07	8362659	5754715
[+] SC-1	2.73E+07	3.04E+07	3.18E+07	1.21E+07	7008866	6084378
[+] SC-2	2.56E+07	2.93E+07	3.02E+07	1.10E+07	6438451	4955168
[+] SC-3	2.52E+07	2.89E+07	2.98E+07	1.13E+07	6067624	5329886
[+] SX-1	3.24E+07	3.52E+07	3.62E+07	1.60E+07	9133866	6103869
[+] SX-2	3.22E+07	3.50E+07	3.57E+07	1.62E+07	9499591	6415780
[+] SX-3	3.34E+07	3.57E+07	3.68E+07	1.69E+07	1.04E+07	7412826

[+] AH-1	3.10E+07	3.49E+07	3.59E+07	1.51E+07	8448925	5004355
[+] AH-2	3.06E+07	3.43E+07	3.53E+07	1.47E+07	7157028	4523457
[+] AH-3	3.16E+07	3.47E+07	3.61E+07	1.47E+07	7619833	4898795
[+] HB-1	0	5132049	6508728	2.09E+07	2.47E+07	2.67E+07
[+] HB-2	5132049	0	490692.1	1.73E+07	2.42E+07	2.82E+07
[+] HB-3	6508728	490692.1	0	1.70E+07	2.44E+07	2.88E+07
[+] GZ-1	2.09E+07	1.73E+07	1.70E+07	0	3638269	6958528
[+] GZ-2	2.47E+07	2.42E+07	2.44E+07	3638269	0	1466623
[+] GZ-3	2.67E+07	2.82E+07	2.88E+07	6958528	1466623	0
